# Efficacy and safety of zhibitai in the treatment of hyperlipidemia: A systematic review and meta-analysis

**DOI:** 10.3389/fphar.2022.974995

**Published:** 2022-09-02

**Authors:** Chunyang Wang, Wentai Pang, Xuechen Du, Jiani Zhai, Mengyuan Zhong, Ming Zhuang, Jiali An, Lujia Cao, Li Zhang, Wenke Zheng, Junhua Zhang

**Affiliations:** ^1^ State Key Laboratory of Component-Based Chinese Medicine, Tianjin University of Traditional Chinese Medicine, Tianjin, China; ^2^ Evidence-Based Medicine Center, Tianjin University of Traditional Chinese Medicine, Tianjin, China; ^3^ Second Teaching Hospital of Tianjin University of Traditional Chinese Medicine, Tianjin, China

**Keywords:** hyperlipidemia, traditional Chinese medicine, zhibitai, systematic review, meta-analysis

## Abstract

**Objective:** To evaluate the efficacy and safety of Zhibitai (ZBT) in the treatment of patients with hyperlipidemia (HLP).

**Methods:** A search of 8 electronic databases was conducted to find randomized controlled trials (RCTs), to evaluate the efficacy and safety of ZBT for the treatment of HLP. The risk of bias in randomized controlled trials was assessed by using the Cochrane Collaboration Risk of Bias tool for randomized controlled trials 2.0 (RoB 2.0). The primary outcomes were the levels of triglyceride (TG), total cholesterol (TC), low-density lipoprotein cholesterol (LDL-C), and high-density lipoprotein cholesterol (HDL-C). The total effective rate served as the secondary outcome. The incidence of adverse events was considered the safety outcome. Review Manager 5.4 was used to conduct meta-analyses. Data were pooled by random-effects or fixed-effects model to obtain the mean difference (MD), risk ratio (RR), odds ratio (OR), and 95% confidence interval (CI).

**Results:** There were 28 eligible RCTs with a total of 2,952 participants. Overall, we verified that ZBT plus conventional therapy (CT) was superior to CT for the treatment of HLP [TC: MD = −0.50, 95% CI (−0.80, −0.19); TG: MD = −0.38, 95% CI (−0.49, −0.27); LDL-C: MD = −0.50, 95% CI (−0.69, −0.31); HDL-C: MD = 0.17, 95% CI (0.11, 0.24); total effective rate: OR = 4.26, 95% CI (2.28, 7.95)]. There were no significant differences in the primary outcomes between ZBT alone vs. CT (*p* > 0.05). For safety, the ZBT group (with CT or alone) outperformed the CT group [ZBT alone: RR = 0.51, 95% CI (0.32, 0.81); ZBT plus CT: RR = 0.51, 95% CI (0.30, 0.89)]. For each outcome, the subgroups and the sensitivity analysis matched the overall results.

**Conclusion:** ZBT may be safe and beneficial to HLP patients, especially for serum lipid management. ZBT can be used along with CT for the treatment of HLP. However, it is necessary to conduct more rigorous RCTs to confirm these findings.

**Systematic Review Registration**: [https://www.crd.york.ac.uk/PROSPERO/], identifier [CRD42022316251].

## 1 Introduction

Hyperlipidemia (HLP) is a common systemic metabolic syndrome associated with lipid abnormalities, including elevated levels of triglyceride (TG), total cholesterol (TC), low-density lipoprotein cholesterol (LDL-C), and reduced high-density lipoprotein cholesterol (HDL-C) levels (Chen et al., 2014; Zhou et al., 2019). HLP has been identified as the primary pathogenic factor in several disorders, including cerebrovascular disease (CVD), diabetes, and kidney dysfunction (Smith, 2007; Karr, 2017; Andreadou et al., 2020; Chen et al., 2020).

In the United States, nearly 53% of adults have elevated LDL-C levels (Centers for Disease and Prevention. 2011). However, only about half of the patients with high LDL-C levels receive therapy, and of those who do, less than three-quarters achieve a satisfactory level of control (Centers for Disease and Prevention. 2011; Mozaffarian et al., 2016). Furthermore, 38.1% of United States adults had total cholesterol levels ≥ 200 mg/dL from 2015 to 2018, which places them at approximately twice the risk of arteriosclerotic cardiovascular disease (ASCVD) as the population with normal total cholesterol levels (Tsao et al., 2022). Adults in China have an HLP prevalence of 40.45% in 2012, which signifies a significant increase over the preceding time (Ding et al., 2015; Joint committee for guideline. 2018; World Health Organization, 2021) Therefore, the prevention and control of HLP are urgently warranted (Stewart et al., 2020).

Statins are the main therapy for HLP (Jacobson et al., 2015; Zhang and Hu, 2015). Numerous studies have convincingly proven the effects of statins on blood lipid profiles (Baigent et al., 2010; Stone et al., 2014; Karr, 2017; Andreadou et al., 2020). Although statins are typically well-tolerated, they are associated with adverse effects, including gastrointestinal reaction, musculoskeletal pain, respiratory infections, and headaches (Karr, 2017; Mollazadeh et al., 2021). Over the past years, traditional Chinese medicine (TCM) has displayed its unique advantages in multi-component, multi-channel and multi-target treatment strategies, making it an excellent complementary and alternative therapy (Gong et al., 2022).

Zhibitai (ZBT) capsule (Chengdu Diao Jiuhong Pharmaceutical Factory, Chengdu, China), an oral Chinese patent medicine, was licensed as a drug used to treat hyperlipidemia by the National Medical Products Administration (NMPA) in May 2019 (NMPA, 2019), including four medicinal herbs. The therapeutic effects of these products related to HLP have been reported: Crataegus pinnatifida Bunge (CPB) [Rosaceae; crataegi fructus] (hypolipidemic effects, anti-inflammatory) (Jurikova et al., 2012), Alisma plantago-aquatica subsp. Orientale (Sam.) Sam. (AR) [Alismataceae; alismatis rhizoma] (anti-hyperlipidemia, antioxidative, anti-inflammatory) (Liu et al., 2020), Atractylodes macrocephala Koidz (AMR) [Asteraceae; atractylodis macrocephalae rhizoma] (neuroprotective effect, anti-inflammatory activity) (Ruqiao et al., 2020), and red yeast rice (RYR) [Rice fermented by monascus] (improving the disorder of lipid metabolism) (Lv et al., 2021). ZBT has the functions of regulating lipid, stabilizing carotid plaque, improving vascular endothelial function and so on, and has few side effects (Su and Hu, 2021). Several clinical studies have shown that ZBT has a good lipid-lowering effect (Xu et al., 2010; Zhao et al., 2017; Xu et al., 2018; Xu et al., 2022). In a mouse model, ZBT can affect the MAPK-ERK-TLRs pathway by down-regulating the expressions of TLR4 and P-ERK1/2 proteins, it can inhibit the transduction of inflammatory signaling pathway, prevent the occurrence and development of inflammation, regulate blood lipids, and antagonize insulin resistance to protect the liver (Liao et al., 2019).

There is a wide acceptance of TCM in China. Alternative therapeutic approaches with complementary therapies are becoming increasingly popular among patients (Nies et al., 2006). Until now, ZBT has been recommended by Chinese expert consensus for HLP treatment and alternative choice of both primary and secondary prophylaxis of cardiovascular diseases (China Heart Federation, 2017). However, the results and methodological quality of existing studies have not been systematically evaluated. Therefore, an evaluation of the efficacy and safety of ZBT in the treatment of HLP was conducted through a systematic review and meta-analysis in the present study.

## 2 Materials and methods

### 2.1 Study registration

The protocol of this systematic review was registered on the PROSPERO (CRD42022316251), and presented according to the guidelines of the Preferred Reporting Items for Systematic Reviews and Meta-Analyses (PRISMA 2020) (Page et al., 2021).

### 2.2 Ethical statement

As this is a literature review, ethical approval was not required.

### 2.3 Information source and search strategy

Eight databases (CNKI, VIP, Wanfang, SinoMed, PubMed, Embase, Cochrane Library, and Web of Science) were searched for randomized controlled trials from inception until March 2022. A manual search of the reference lists of the included trials was also conducted to determine the additional relevant studies. The research strategy of PubMed was as follows:

#1 Hyperlipidemias [MeSH Terms]
#2 Hyperlipemi [Title/Abstract]
#3 Hyperlipemias [Title/Abstract]
#4 hyperlipidemia [Title/Abstract]
#5 Lipidemia [Title/Abstract]
#6 Lipidemias [Title/Abstract]
#7 Lipemia [Title/Abstract]
#8 Lipemias [Title/Abstract]
#9 #1 OR #2 OR #3 OR #4 OR #5 OR #6 OR #7 OR #8
#10 Zhibitai [Title/Abstract]
#11 #9 AND #10

### 2.4 Inclusion criteria

#### 2.4.1 Type of study

Randomized controlled trials (RCTs) and articles published only in the English and Chinese languages were considered.

#### 2.4.2 Participants

Hyperlipidemia was defined according to the Guidelines for the Prevention and Treatment of Dyslipidemia in Chinese Adults (as revised in 2016) (Joint committee issued Chinese guideline for the management of dyslipidemia in adults. 2016) and the 2019 ESC/EAS guidelines (Mach et al., 2020). The patients of hyperlipidemia included four types, classified according to the following clinical classification: 1) hypercholesterolemia: TC ≥ 5.2 mmol/L and TG ≤ 1.7 mmol/L; 2) hypertriglyceridemia: TG ≥ 1.7 mmol/L and TC ≤ 5.2 mmol/L; 3) mixed hyperlipidemia: TC ≥ 5.2 mmol/L and TG ≤ 1.7 mmol/L; 4) low high-density lipoproteinemia: HDL-C ≤ 1.0 mmol/L, age and nationality were unrestricted.

#### 2.4.3 Intervention and comparison

The participants were treated with conventional therapy (CT), including atorvastatin, pitavastatin calcium, rosuvastatin, simvastatin, fenofibrate, Fluvastatin and symptomatic treatment were assigned to the control group. In comparison, the intervention group was administered with only ZBT or ZBT plus the control group, irrespective of the dose, and the duration or frequency of administration of ZBT. CT was administered to the intervention group to the same manner as in the control group.

#### 2.4.4 Outcomes

1) Primary outcomes: TC, TG, LDL-C, and HDL-C levels.
2) Secondary outcome: total effective rate.

Significant Effective: TG decreased by > 40%, TC decreased by > 20% or HDL-C increased by > 0.26 mmol/L; Effective: TG decreased by 20%–40%, TC decreased by 10%–20%, or HDL-C increased by 0.1–0.26 mmol/L; Invalid: the above standards were not met (Zhao, 2007). Total effective rate = number of effective cases/total number of cases × 100%.

3) Safety Outcome: The incidence of adverse events (AEs), including gastrointestinal reaction, muscular pain, or respiratory infections.

### 2.5 Exclusion criteria

1) Incorrect or incomplete data.
2) Publications that were repeated, with only the latest or largest articles included.
3) Patients who were combined with other diseases, such as chronic diseases, and metabolic diseases were excluded.

### 2.6 Study screening and data extraction

The study was screened as per the following scheme: 1) the titles and abstracts of the manuscripts were read and the eligible studies were included as per the inclusion criteria; 2) for the screening process, if more information was required, the full text was examined.

To extract the data, a standard extraction sheet was created that included the following: study title, author, study duration, sample size, age and gender of patients, diagnosis, duration of disease, treatment measures, follow-up, and outcomes. If the complete data could not be obtained, the author was contacted *via* email. If the author failed to respond, the study was excluded. Two researchers independently screened and extracted the data from the study. Any disagreements were resolved through consensus or by consulting with a third researcher.

### 2.7 Quality assessment

In each study, quality assessment was performed by two investigators using the Cochrane Collaboration Risk of Bias tool for randomized controlled trials 2.0 (RoB 2.0). An assessment of bias was made based on specified signaling questions for each of the following 5 domains: 1) randomization process, 2) deviations from the intended interventions, 3) missing outcome data, 4) measurement of the outcome, and 5) selection of the reported outcome. For each domain, the risk of bias was characterized as “low risk,” “some concerns,” or “high risk”. Based on the risk of bias for each domain, the overall risk of bias judgment was prepared for each study (Sterne et al., 2019).

### 2.8 Statistical analysis

A meta-analysis was conducted using the Review Manager software (Cochrane Collaboration, version 5.4). The continuous data were expressed as the mean difference (MD), while the dichotomous data were expressed as the risk ratio (RR) or odds ratio (OR). The 95% confidence intervals (CI) were applied. The I^2^ statistic and the Chi2 test were used to assess statistical heterogeneity. Significant heterogeneity was presented when *I* ^*2*^ > 50% or *p* < 0.1, first, the cause of the heterogeneity was established by running a sensitivity analysis to eliminate the likely source of the heterogeneity. If the heterogeneity was still significant, the random-effect model was used to accommodate heterogeneity (Shi et al., 2022). Conversely, the fixed-effect model was applied in the absence of substantial heterogeneity (*I* ^*2*^ ≤ 50% or *p* ≥ 0.1) (Higgins et al., 2003). Two-tailed *p* < 0.05 was considered to indicate statistical significance. A subgroup analysis of the study results was conducted based on the dosage and duration of the intervention in order to investigate the possible causes of heterogeneity. A sensitivity analysis of the merged results was performed by eliminating the individual studies. The funnel plots were evaluated to determine the potential publication bias in a meta-analysis when at least 10 studies were deemed eligible for inclusion.

### 2.9 Certainty of evidence

With the GRADE (Grading of Recommendation, Assessment, Development, and Evaluation) tool, the certainty of the evidence and strength of the recommendation were assessed (Meerpohl et al., 2012). The results were summarized using the GRADE pro-GDT (http://gdt.guidelinedevelopment.org). A variety of factors were considered during this evaluation, which included the study design, methodological limitations, inconsistencies, indirect evidence, imprecision, and other factors. Depending on the level of evidence, the quality was considered to be either high, moderate, low or very low.

## 3 Results

### 3.1 Study screening

**FIGURE 1:**
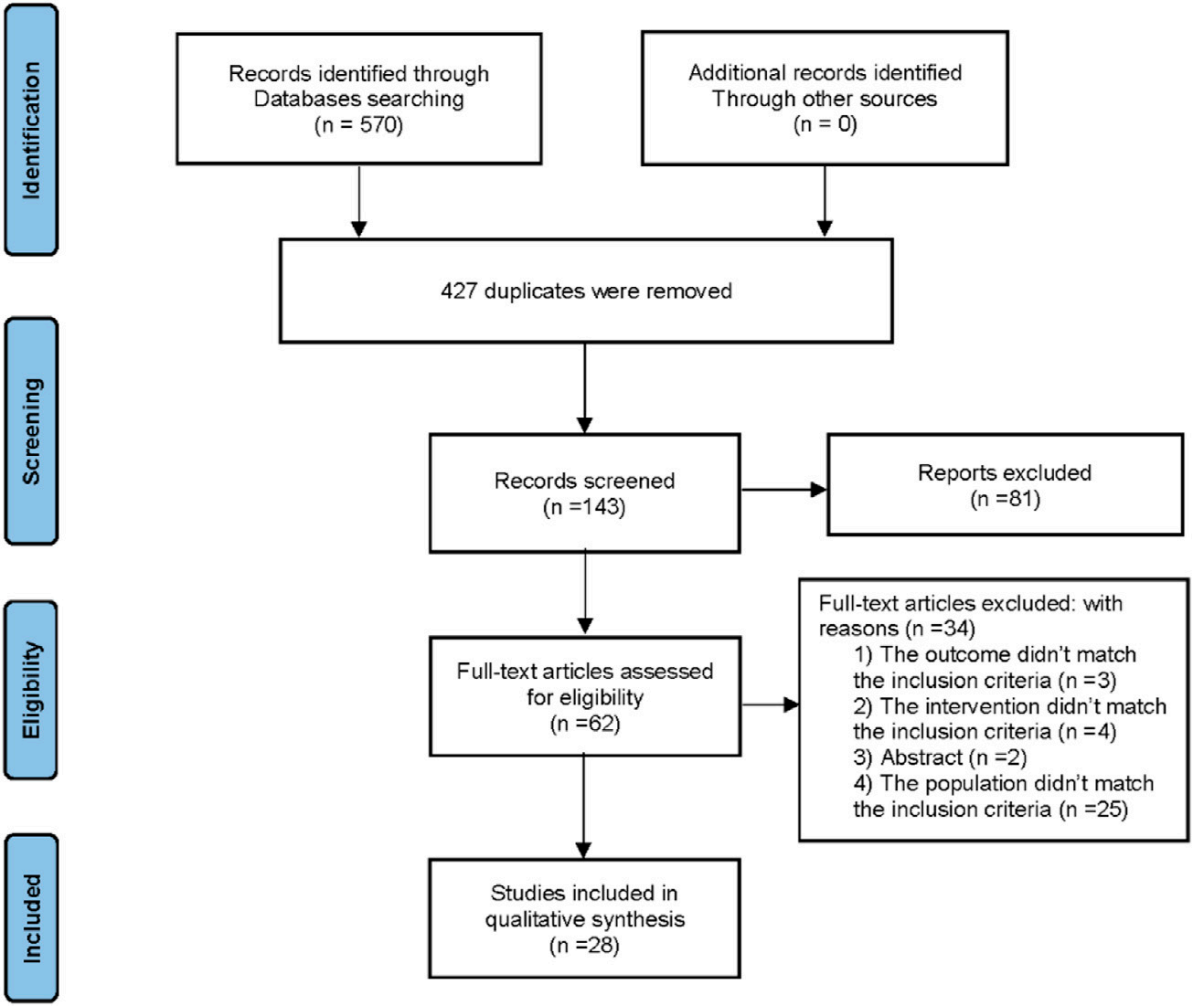
Flow diagram depicting the study screening process.

A total of 570 records were retrieved from all databases and 427 records were eliminated as they were duplicates. After a review of the titles and abstracts, 81 more articles were eliminated, which left 62 records for further assessment. After reviewing the full text, 34 articles were excluded due to the following reasons: 1) The outcome did not meet the inclusion criteria (*n* = 3); 2) The intervention did not meet the inclusion criteria (*n* = 4); 3) Abstract (*n* = 2); 4) The population did not meet the inclusion criteria (*n* = 25). Finally, this systematic review comprised 28 articles (Li, 2007; Xu et al., 2010; Zhu and Ma. 2010; Liu and Zhang. 2011; Hu and Li, 2012; Zhou et al., 2012; Hang et al., 2013; Hao, 2013; He, 2013; Xiang, 2013; Zhao, 2013; Sun, 2014; Zhou, 2014; Wang and Chen, 2015; Chen et al., 2016; Bai, 2018; Ma and Feng, 2018; Pang, 2018; Bai, 2019; Shi, 2019; Xiong, 2019; Chen et al., 2020; He, 2020; Hua, 2020; Li, 2020; Xiao, 2020; Pan, 2021; Tan et al., 2021). Figure 1 depicts the screening process for the study.

### 3.2 Study characteristics

Table 1 provides an overview of all study characteristics. All 28 included studies were RCTs published between 2007 and 2021. The 28 RCTs enrolled a total of 2,952 participants. Eleven RCTs with 1,199 participants compared ZBT plus CT with CT, 17 RCTs with 1,753 participants compared ZBT alone with CT. CT include atorvastatin, pitavastatin calcium, rosuvastatin, simvastatin, and fenofibrate. The follow-up period varied from 4 to 48 weeks.

**TABLE 1:** Basic characteristics of the included studies.

Study	Disease	Year (T/C)	Sample size (T/C)	Intervention		Course of treatment (weeks)	Outcome	Adverse event (T/C)
				T (dosage)	C			
Li (2007)	HLP	32–78/30–80	37/39	ZBT (480 mg)	CT (a)	4	①②③④	T: None
								C: abnormal liver function
Xu et al. (2010)	HLP	63.51 ± 4.57/64.08 ± 4.08	78/79	ZBT (480 mg)	CT (a)	8	①②③④	Not mentioned
Zhu and Ma. (2010)	HLP	74.2 ± 8.1/72.8 ± 8.9	60/58	ZBT (960 mg)	CT (e)	12	①②③④	Not mentioned
Liu and Zhang (2011)	HLP	—	60/60	ZBT (480 mg)	CT (a)	8	①②③④	Not mentioned
Hu and Li. (2012)	HLP	51.3	79/79	ZBT (480 mg)	CT (f)	4	①②③④⑤	Not mentioned
Zhou et al. (2012)	HLP	59.13 ± 9.20/61.42 ± 8.52	69/70	ZBT (480 mg)	CT (e)	48	①②③④	Not mentioned
Hao (2013)	HLP	61.77/64.92	47/47	ZBT (480 mg)	CT (a)	9	①②③	T: gastrointestinal reaction;
								C: increase abnormally in the level of CK; gastrointestinal reaction; abnormal liver function erythra
He (2013)	HLP	48.2 ± 13.5/47.2 ± 14.4	44/44	ZBT (480 mg)	CT (e)	8	①②③④⑤	T: None
								C: gastrointestinal reaction, myodynia
Hang et al. (2013)	HLP	45–81	50/50	ZBT (480 mg)	CT (f)	12	①②③④	T/C: gastrointestinal reaction, myodynia, abnormal liver function,
Xiang (2013)	HLP	—	40/40	ZBT (480 mg)	CT (a)	6	⑤	T: gastrointestinal reaction, myodynia
								C: myodynia, abnormal liver function
Zhao (2013)	HLP	60–82/60–83	73/73	ZBT (480 mg) +C	CT (a)	16	⑤	Not mentioned
Sun (2014)	HLP	72.11 ± 0.23/71.65 ± 0.58	33/33	ZBT (480 mg) +C	CT (a)	12	①②③④⑤	T/C: gastrointestinal reaction
Zhou (2014)	HLP	—	60/60	ZBT (480 mg)	CT (e)	48	①②③④	Not mentioned
Wang and Chen (2015)	HLP	66.5 ± 5.2/67.2 ± 4.5	32/32	ZBT (480 mg)	CT (a)	6	①②③④	T/C: gastrointestinal reaction
Chen et al. (2016)	HLP	62.5 ± 5.6/63.5 ± 6.2	42/42	ZBT (480 mg) +C	CT (a)	8	①②③④⑤	Not mentioned
Bai (2018)	HLP	56.4 ± 2.1/55.1 ± 2.6	75/75	ZBT (960 mg)	CT (e)	8	①②③	T/C: gastrointestinal reaction, abnormal liver function, erythra
Ma and Feng (2018)	HLP	18–75	29/28	ZBT (480 mg) + C	CT (d)	8	①②③④	T/C: increase abnormally in the level of CK, gastrointestinal reaction
Pang (2018)	HLP	45–85/42–85	30/30	ZBT (480 mg)	CT (e)	4	①②③	Not mentioned
Bai (2019)	HLP	—	34/33	ZBT (480 mg)	CT (a)	6	①②③④	Not mentioned
Shi (2019)	HLP	52.63 ± 5.42/52.59 ± 5.39	65/65	ZBT (480 mg) + C	CT (e)	8	①②③④⑤	Not mentioned
Xiong (2019)	HLP	66.32 ± 2.21/66.28 ± 2.18	45/45	ZBT (960 mg) + C	CT (e)	8	①②③④	Not mentioned
Chen et al. (2020)	HLP	70.1 ± 7.6/71.2 ± 8.1	63/63	ZBT (480 mg) + C	CT (a)	12	①②③④⑤	T/C: gastrointestinal reaction
He (2020)	HLP	54.86 ± 10.08/55.01 ± 11.32	80/80	ZBT (960 mg) + C	CT (b)	8	①②③④	T/C: gastrointestinal reaction
Hua (2020)	HLP	52–71/51–72	40/40	ZBT (480 mg)	CT (a)	8	①②③④	T/C: gastrointestinal reaction
Li (2020)	HLP	41–68/43–70	41/41	ZBT (960 mg)	CT (a)	8	①②③④⑤	T: gastrointestinal reaction
								C:abnormal liver function
Xiao (2020)	HLP	71.5 ± 2.4/72.3 ± 2.7	60/60	ZBT (960 mg) + C	CT (a)	8	①②③④	T/C: gastrointestinal reaction, abnormal liver function
Pan (2021)	HLP	55.83 ± 9.37/55.37 ± 9.28	50/50	ZBT (480 mg) + C	CT (d)	4	⑤	T/C: gastrointestinal reaction, myodynia, dizziness
Tan et al. (2021)	HLP	83.05 ± 1.52/84.25 ± 0.75	60/60	ZBT (480 mg) + C	CT (d)	4	①②③④	T/C: palpitation, flushed complexion;

Abbreviations: T, treatment group; C, control group; ZBT, zhibitai capsule; CT, conventional therapy; a, atorvastatin; b, pitavastatin calcium; d, rosuvastatin; e, simvastatin; f, fenofibrate; ①, TC; ②, TG; ③, LDL-C; ④, HDL-C; ⑤, total effective rate.

### 3.3 Quality assessment

**FIGURE 2:**
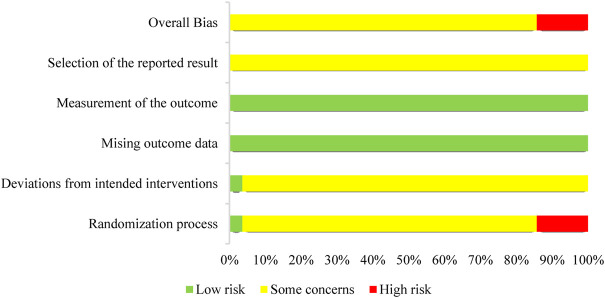
Risks of bias of the included studies. Each included study’s bias risk was examined by the authors. Red, high risk; yellow, some concerns; green, low risk.

Four studies showed significant bias from the randomization process (Zhao, 2013; Bai, 2018; Shi, 2019; Xiao, 2020), 1 study (Xu et al., 2010) had a low bias and 23 studies had some bias. A total of 27 papers exhibited some bias due to deviations from targeted interventions, 1 study (Xu et al., 2010) showed a low bias. Twenty-eight studies papers exhibited minimal bias in the domain of bias due to missing outcome data. In the domain of bias in outcome measurement, 28 studies exhibited low bias. Twenty-eight studies demonstrated some bias in the domain bias of selection of the reported outcome. Overall, the risk of bias assessment ranged from “some” to “high” across the included studies. Figure 2 depicts an overview of the methodological quality assessment results.

### 3.4 Meta-analysis results

#### 3.4.1 Cholesterol

**FIGURE 3:**
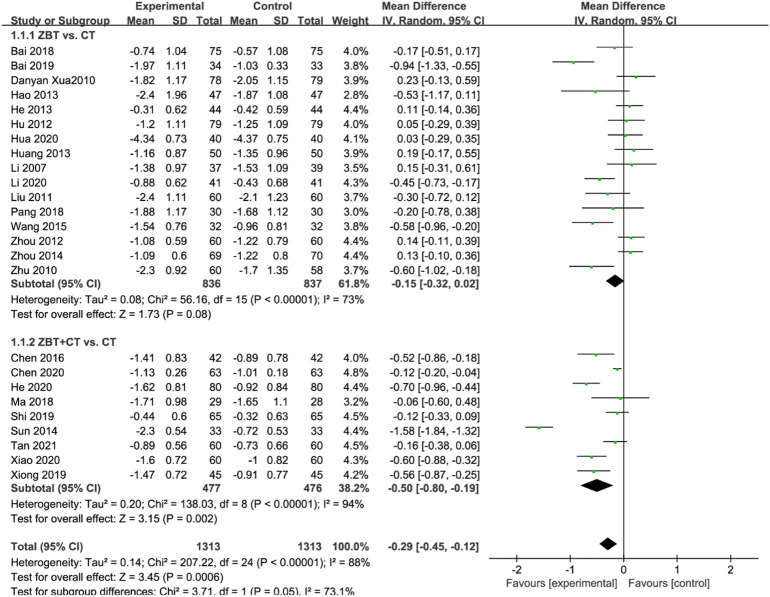
Meta-analysis results of the effect of Zhibitai (ZBT) vs. conventional therapy (CT) on total cholesterol (TC) level.

TC was reported in 25 RCTs (2,626 participants) (Li, 2007; Xu et al., 2010; Zhu and Ma. 2010; Liu and Zhang. 2011; Hu and Li, 2012; Zhou et al., 2012; Hang et al., 2013; Hao, 2013; He, 2013; Sun, 2014; Zhou. 2014; Wang and Chen, 2015; Chen et al., 2016; Bai, 2018; Ma and Feng, 2018; Pang, 2018; Bai, 2019; Shi, 2019; Xiong, 2019; Andreadou et al., 2020; Chen et al., 2020; He, 2020; Hua, 2020; Li, 2020; Xiao, 2020; Tan et al., 2021) (Figure 3). Significant heterogeneity was noted (*I* ^*2*^ = 88%). After analyzing the sensitivity and removing each piece of literature for the heterogeneity test, the findings revealed that the heterogeneity was still significant; accordingly, the random effects model was utilized. Subgroup analyses were performed according to the interventions (ZBT alone or ZBT plus CT). In the subgroup of ZBT vs. CT (16 RCTs including 1,673 participants), no significant difference was noted between the two groups [MD = −0.15, 95% CI (−0.32, 0.02), *p* = 0.08], suggesting that ZBT may be equally effective as CT in reducing TC. In the subgroup of ZBT plus CT vs. CT (9 RCTs including 953 participants), this meta-analysis revealed a significant improving effect of ZBT plus CT on TC [MD = −0.50, 95% CI (−0.80, −0.19), *p* < 0.0001]. The result indicated that ZBT might improve the efficacy of CT in reducing TC.

#### 3.4.2 Triglyceride

**FIGURE 4:**
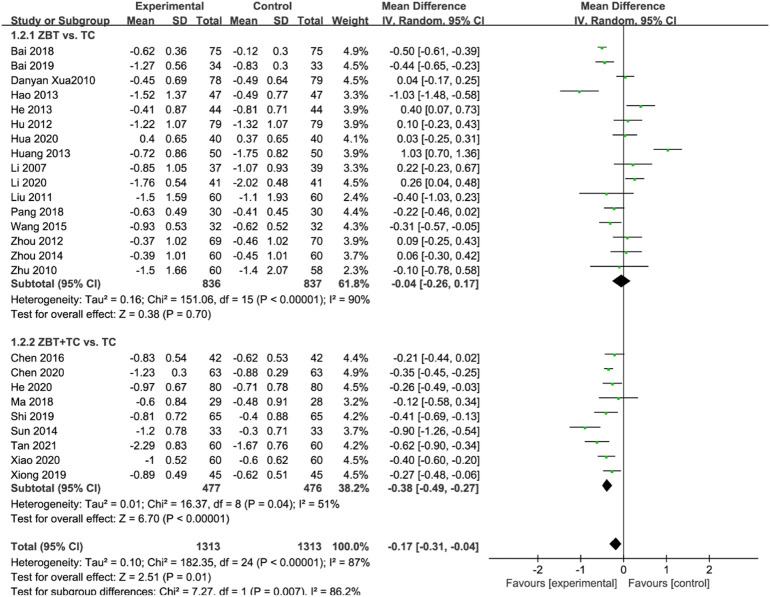
Meta-analysis results of the effect of Zhibitai (ZBT) vs. conventional therapy (CT) on triglyceride (TG).

TG was reported in 25 RCTs (2,626 participants) (Li. 2007; Xu et al., 2010; Zhu and Ma. 2010; Liu and Zhang. 2011; Hu and Li. 2012; Zhou et al., 2012; Hang et al., 2013; Hao. 2013; He. 2013; Sun. 2014; Zhou. 2014; Wang and Chen. 2015; Chen., 2016; Bai. 2018; Ma and Feng. 2018; Pang, 2018; Bai. 2019; Shi. 2019; Xiong. 2019; Chen et al., 2020; He. 2020; Hua. 2020; Li. 2020; Xiao. 2020; Pan. 2021; Tan et al., 2021) (Figure 4). Significant heterogeneity was noted (*I* ^*2*^ = 87%). The sensitivity was analyzed, and the literature was excluded one by one for the heterogeneity test. The results revealed that the heterogeneity was still large, and therefore the random effects model was applied. Subgroup analyses were performed according to the interventions (ZBT alone or ZBT plus CT). In the subgroup of ZBT vs. CT (16 RCTs including 1,673 participants), no significant difference was noted between the two groups [MD = −0.04, 95% CI (−0.26, 0.17), *p* = 0.70], suggesting that ZBT may be equally effective as CT in reducing TG. In the subgroup of ZBT plus CT vs. CT (9 RCTs including 953 participants), the group of ZBT plus CT significantly improve the level of TG [MD = −0.38, 95% CI (−0.49, −0.27), *p* < 0.0001]. The result implied that ZBT may improve the efficacy of CT in reducing TG.

#### 3.4.3 Low-density lipoprotein cholesterol

**FIGURE 5:**
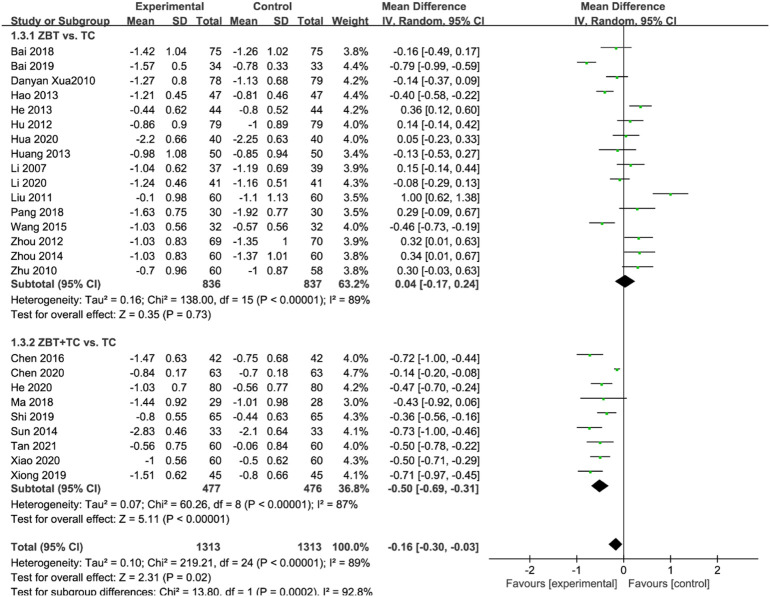
Meta-analysis results of the effect of Zhibitai (ZBT) vs. conventional therapy on low-density lipoprotein cholesterol (LDL-C).

LDL-C levels were reported in 25 RCTs (2,626 participants) (Li, 2007; Xu et al., 2010; Zhu and Ma, 2010; Liu and Zhang, 2011; Hu and Li. 2012; Zhou et al., 2012; Hang et al., 2013; Hao, 2013; He, 2013; Sun, 2014; Zhou, 2014; Wang and Chen. 2015; Chen et al., 2016; Bai, 2018; Ma and Feng, 2018; Pang, 2018; Bai, 2019; Shi, 2019; Xiong, 2019; Chen et al., 2020; He, 2020; Hua, 2020; Li, 2020; Li and Li, 2020; Xiao, 2020; Tan et al., 2021) (Figure 5). Significant heterogeneity was noted (*I* ^*2*^ = 89%). The sensitivity was analyzed, and the literature was excluded one by one for the heterogeneity test. The results implied that the heterogeneity was still large, therefore the random effects model was applied. Subgroup analyses were performed according to the interventions (ZBT alone or ZBT plus CT). In the subgroup of ZBT vs. CT (16 RCTs including 1,673 participants), no significant difference was noted between the two groups [MD = 0.04, 95% CI (−0.17, 0.24), *p* = 0.73], suggesting that ZBT may be equally effective as CT in reducing LDL-C. Different results were recorded in the subgroup of ZBT plus CT vs. CT [MD = −0.50, 95% CI (−0.69, −0.31), *p* < 0.0001], suggesting which implied that ZBT may improve the efficacy of CT in reducing LDL-C.

#### 3.4.4 High-density lipoprotein cholesterol

**FIGURE 6:**
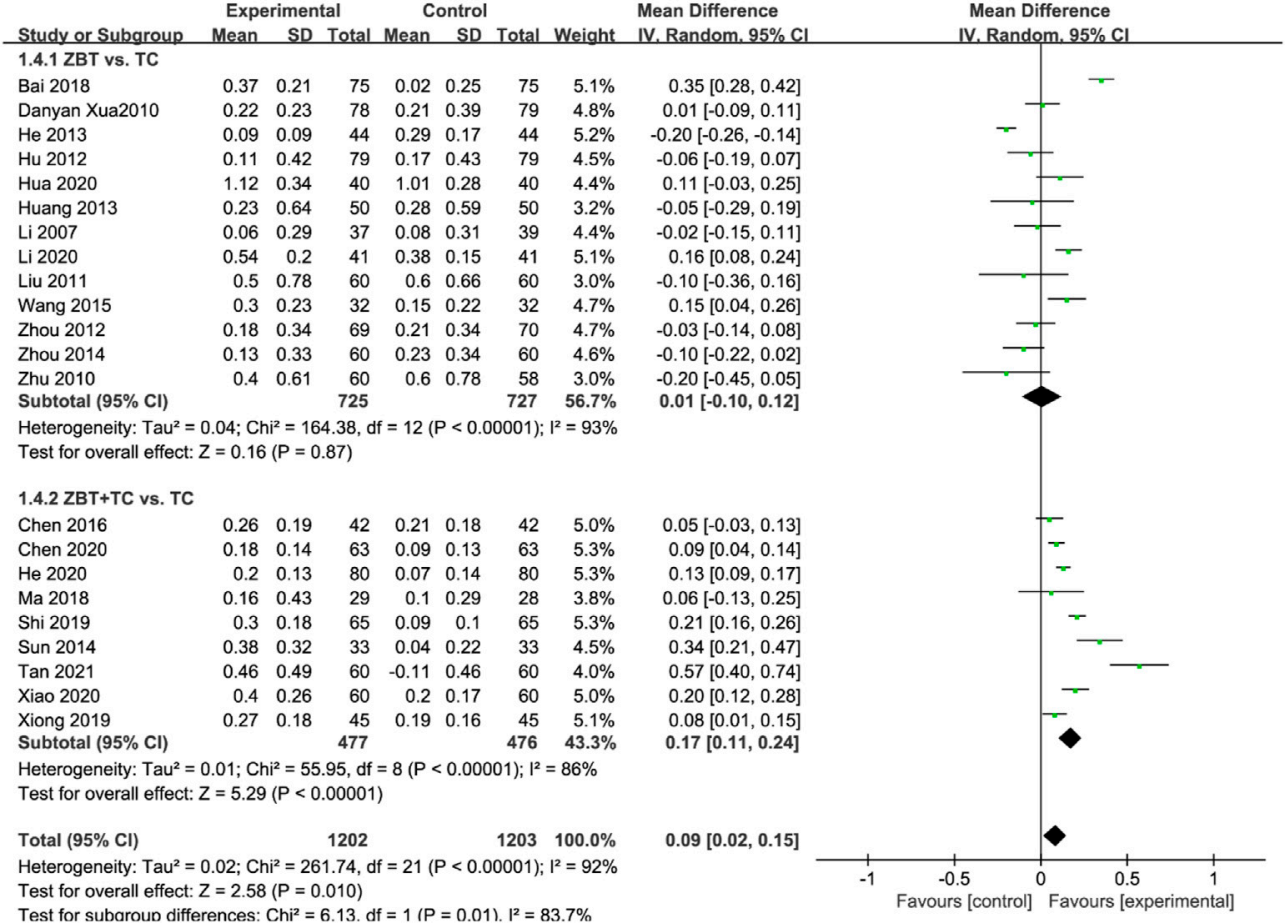
Meta-analysis results of the effect of Zhibitai (ZBT) vs. conventional therapy (CT) on high-density lipoprotein cholesterol (HDL-C).

HDL-C was reported in 22 RCTs (2,405 participants) (Li, 2007; Xu et al., 2010; Zhu and Ma, 2010; Liu and Zhang, 2011; Hu and Li, 2012; Zhou et al., 2012; Hang et al., 2013; He, 2013; Zhao, 2013; Sun., 2014; Zhou, 2014; Wang and Chen, 2015; Chen et al., 2016; Bai, 2018; Ma and Feng, 2018; Shi, 2019; Xiong, 2019; Chen et al., 2020; He, 2020; Hua, 2020; Li, 2020; Xiao, 2020; Tan et al., 2021) (Figure 6). Significant heterogeneity was noted (*I* ^*2*^ = 92%). The sensitivity was analyzed, and the literature was excluded one by one for the heterogeneity test. The results implied that the heterogeneity was still large, therefore the random effects model was adopted. Subgroup analyses were performed according to the interventions (ZBT alone or ZBT plus CT). In the subgroup of ZBT vs. CT (13 RCTs including 1,452 participants), no significant difference was noted between the two groups [MD = 0.01, 95% CI (−0.10, 0.12), *p* = 0.87], suggesting that ZBT may be equally effective as CT in improving HDL-C. In the subgroup of ZBT plus CT vs. CT, the group of ZBT plus CT may significantly improve the efficacy of CT in improving HDL-C [MD = 0.17, 95% CI (0.11, 0.24), *p* < 0.0001], suggesting that ZBT might improve the efficacy of CT in improving HDL-C.

#### 3.4.5 Total effective rate

**FIGURE 7:**
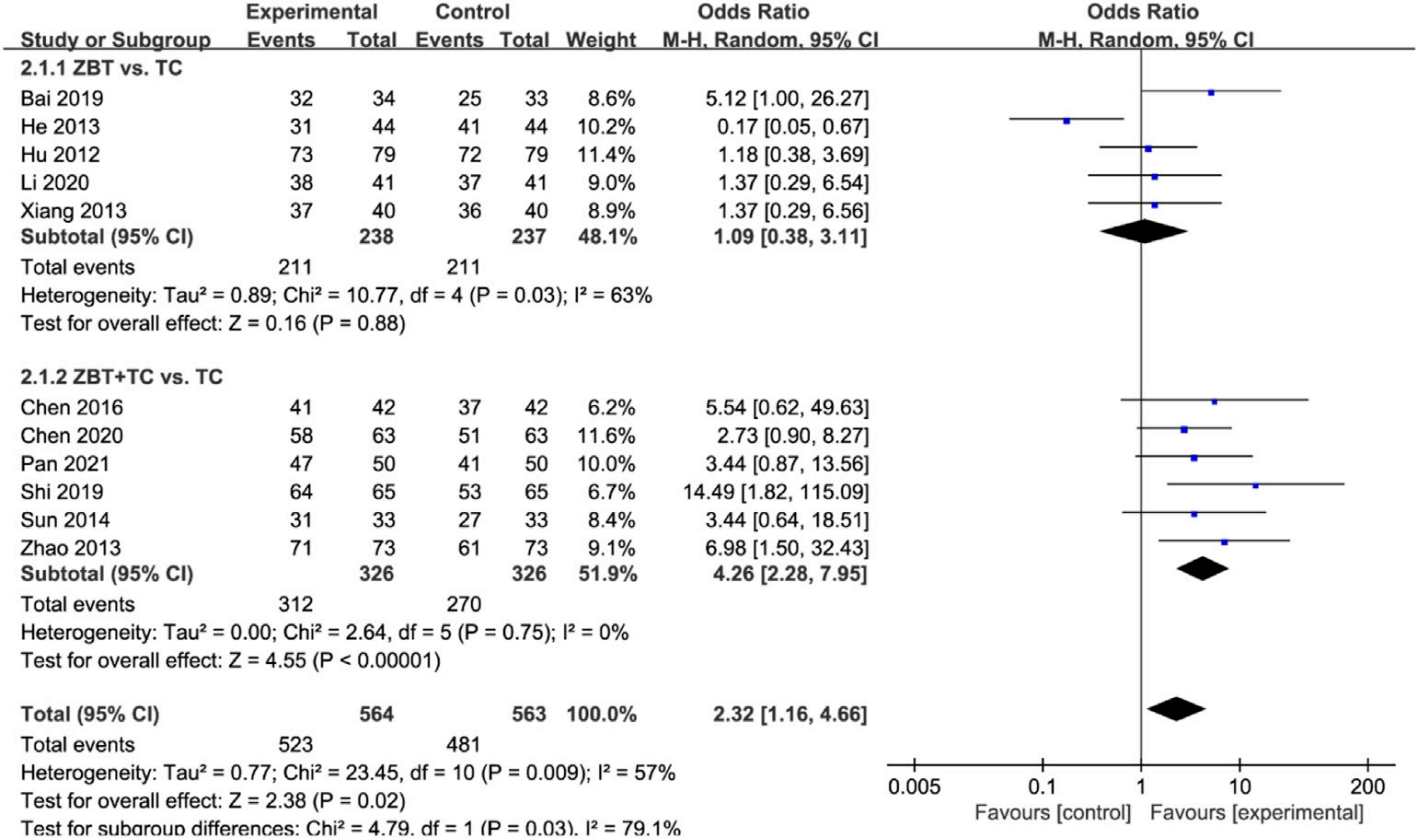
Meta-analysis results of the effect of Zhibitai (ZBT) vs. conventional therapy (CT) on total effective rate.

The total effective rate was reported in 11 RCTs (1,004 participants) (Hu and Li. 2012; He, 2013; Xiang, 2013; Zhao, 2013; Sun, 2014; Wang and Chen, 2015; Chen et al., 2016; Bai, 2019; Shi, 2019; Chen et al., 2020; Li, 2020; Pan, 2021) (Figure 7). Significant heterogeneity was noted (*I* ^*2*^ = 92%). The sensitivity was analyzed, and the literature was excluded one by one for the heterogeneity test. The results show that the heterogeneity was still large, therefore the random effects model was adopted. Subgroup analyses were performed according to the interventions (ZBT alone or ZBT plus CT). In the subgroup of ZBT vs. CT (5 RCTs including 475 participants), there was no significant difference between the two groups [OR = 1.09, 95% CI (0.38, 3.11), *p* = 0.88], suggesting that ZBT may be equally effective as CT in improving the total effective rate. Similar results were recorded in the subgroup of ZBT plus CT vs. CT [OR = 4.26, 95% CI (2.28, 7.95), *p* < 0.0001], implying that ZBT may improve CT efficacy in improving the total effective rate.

### 3.5 Adverse events

There were 16 studies (Li, 2007; Hang et al., 2013; Hao, 2013; He, 2013; Xiang, 2013; Sun, 2014; Wang and Chen, 2015; Bai, 2018; Ma and Feng, 2018; Chen et al., 2020; He, 2020; Hua, 2020; Li. 2020; Xiao, 2020; Wu et al., 2020; Pan, 2021; Tan et al., 2021) that documented adverse events during the treatment. Other studies did not report AEs. The major AEs were gastrointestinal reactions, myodynia, abnormal liver function, and erythema (Tables 2, 3).

The incidence of any AEs was 5.91% (24/406) in the ZBT group alone and 11.76% (48/408) in CT group [RR = 0.51, 95% CI (0.32, 0.81), *p* = 0.004]. The result showed that the ZBT group alone had significantly fewer AEs than the CT group. The ZBT alone group had significantly fewer abnormal liver functions than the CT group [RR = 0.40, 95% CI (0.18, 0.90), *p* = 0.03]. The two groups performed similarly for gastrointestinal reactions, myodynia, erythema, and other AEs (*p* > 0.05) (Table 2).

**TABLE 2:** Subgroup analysis of the main safety outcomes of ZBT vs. CT.

Safety outcomes	No. S	RR	95% CI	*I* ^*2*^	*p* value
Any AEs	9	0.51	(0.32–0.81)	0%	0.004
Gastrointestinal reaction AEs	8	0.62	(0.30–1.28)	0%	0.20
Abnormal liver function AEs	6	0.40	(0.18–0.90)	0%	0.03
Myodynia AEs	3	1.00	(0.23–4.32)	0%	1.00
Erythema AEs	1	0.58	(0.21–1.59)	0%	0.29
Other AEs	1	0.33	(0.01–7.98)	/	0.50

Abbreviations: No. S, numbers of studies; CT, conventional therapy; CI, confidence interval; *I* ^*2*^, heterogeneity; RR, risk ratio; AEs, adverse events; Other AEs included dizziness, fever, heart palpitations, headache, fatigue, cough; /, not applicable.

The incidence of any AEs was 4.80% (18/375) in ZBT plus CT group and 9.36% (35/374) in CT group [RR = 0.51, 95% CI (0.30, 0.89), *p* = 0.02]. The result showed significantly fewer AEs occurred in the ZBT plus CT group when compared with those in the CT group. The two groups performed similarly for gastrointestinal reactions, abnormal liver function, myodynia, and other AEs (*p* > 0.05) (Table 3).

**TABLE 3:** Subgroup analysis of the main safety outcomes of ZBT + CT vs. CT.

Safety outcomes	No. S	RR	95% CI	*I* ^*2*^	*p* value
Any AEs	7	0.51	(0.30–0.89)	0%	0.02
Gastrointestinal reaction AEs	6	0.55	(0.27–1.12)	0%	0.10
Abnormal liver function AEs	1	1.00	(0.06–15.62)	/	1.00
Myodynia AEs	1	0.33	(0.04–3.10)	0%	0.33
Other AEs	3	0.45	(0.16–1.27)	0%	0.13

Abbreviations: No. S, numbers of studies; CT, conventional therapy; CI, confidence interval; *I* ^*2*^, heterogeneity; RR, risk ratio; AEs, adverse events; Other AEs included dizziness, fever, heart palpitations, headache, fatigue, cough; /, not applicable.

### 3.6 Sensitivity analyses

Based on the sensitivity analyses, the results of the two types of interventions were similar to the pooled results. The meta-analysis results did not change when any study was deleted, suggesting that the results were stable. The data for sensitivity analysis are shown in Supplementary Appendix SI.

### 3.7 Subgroup analysis

The subgroup analysis of two interventions for each efficacy outcome was conducted based on the treatment duration (<8 weeks, ≥8 weeks), and ZBT dosage (480, 960 mg). The results of each subgroup analysis were consistent with the overall results (Tables 4, 5). As for ZBT vs. CT, the difference in interactive effect was significant in TC with different dosages of ZBT (480 mg: MD = −0.09; 95% CI −0.27 to 0.10; *I* ^*2*^ = 72%; 960 mg: MD = −0.39; 95% CI −0.62 to −0.16; *I* ^*2*^ = 28%; *p*_interaction_ of dosages = 0.04). As for ZBT plus vs. CT, the difference in interactive effect was significant in HDL-C with different treatment duration (≤8 weeks: MD = 0.57; 95% CI 0.40 to 0.74; >8 weeks: MD = 0.14; 95% CI 0.09 to 0.19; *I* ^*2*^ = 78%; *P*_interaction_ of duration < 0.001).

**TABLE 4:** Subgroup analysis of the main efficacy outcomes of ZBT vs. CT.

Subgroup		No. S	MD/OR	95% CI	*I* ^*2*^ (%)	*P*_interaction_
Different treatment duration						
TG	<8 weeks	5	−0.18	−0.39 to 0.04	66	0.35
	≥8 weeks	11	0	−0.31 to 0.31	93	
TC	<8 weeks	5	−0.31	−0.73 to 0.11	80	0.31
	≥8 weeks	11	−0.07	−0.24 to 0.09	65	
LDL-C	<8 weeks	5	−0.15	−0.59 to 0.30	92	0.29
	≥8 weeks	11	0.12	−0.10 to 0.34	85	
HDL-C	<8 weeks	3	0.03	−0.11 to 0.16	71	0.81
	≥8 weeks	10	0	−0.14 to 0.15	94	
Total effective rate	<8 weeks	3	1.78	0.76 to 4.17	10	0.23
	≥8 weeks	2	0.47	0.06 to 3.54	74	
Different dosages of ZBT						
TG	480 mg	13	−0.02	−0.26 to 0.21	87	0.77
	960 mg	3	−0.12	−0.73 to 0.49	95	
TC	480 mg	13	−0.09	−0.27 to 0.10	72	0.04
	960 mg	3	−0.39	−0.62 to −0.16	28	
LDL-C	480 mg	13	0.04	−0.21 to 0.30	91	0.84
	960 mg	3	0.01	−0.24 to 0.26	56	
HDL-C	480 mg	10	−0.03	−0.11 to 0.06	81	0.14
	960 mg	3	0.14	−0.07 to 0.35	92	
Total effective rate	480 mg	4	1.04	0.28 to 3.90	72	0.79
	960 mg	1	1.37	0.29 to 6.54	63	

Abbreviations: No. S, numbers of studies; MD, mean difference; OR, odds ratio; *P*_interaction_, *P* for interaction; *I* ^*2*^, heterogeneity; ZBT, zhibitai capsules; HLP, hyperlipidemia; Comorbid Diseases, Hyperlipidemia complicated by chronic disease; CT, conventional therapy; /, not applicable.

**TABLE 5:** Subgroup analysis of the main efficacy outcomes of ZBT + CT vs. CT.

Subgroup		No. S	MD/OR	95% CI	*I* ^*2*^	*P*_interaction_
Different treatment duration						
TG	<8 weeks	1	−0.62	−0.91 to −0.33	/	0.07
	≥8 weeks	8	−0.34	−0.41 to −0.27	46%	
TC	<8 weeks	1	−0.16	−0.38 to −0.06	/	0.08
	≥8 weeks	8	−0.54	−0.89 to −0.18	95%	
LDL-C	<8 weeks	1	−0.50	−0.79 to −0.21	/	0.99
	≥8 weeks	8	−0.50	−0.70 to −0.29	88%	
HDL-C	<8 weeks	1	0.57	0.40 to 0.74	/	<0.001
	≥8 weeks	8	0.14	0.09 to 0.19	78%	
Total effective rate	<8 weeks	1	3.44	0.87 to 13.56	/	0.64
	≥8 weeks	5	4.98	2.51 to 9.85	0%	
Different dosages of ZBT						
TG	480 mg	6	−0.43	−0.60 to −0.25	66%	0.30
	960 mg	3	−0.31	−0.44 to −0.19	0%	
TC	480 mg	6	−0.43	−0.85 to −0.01	96%	0.39
	960 mg	3	−0.63	−0.79 to −0.47	0%	
LDL-C	480 mg	6	−0.47	−0.71 to −0.22	87%	0.59
	960 mg	3	−0.54	−0.68 to −0.41	4%	
HDL-C	480 mg	6	0.21	0.10 to 0.32	90%	0.24
	960 mg	3	0.13	0.08 to 0.19	59%	
Total effective rate	480 mg	6	4.65	2.52 to 8.56	0%	<0.001

Abbreviations: No. S, numbers of studies; MD, mean difference; OR, odds ratio; *P*_interaction_, *P* for interaction; *I* ^*2*^, heterogeneity; ZBT, zhibitai capsules; HLP, hyperlipidemia; Comorbid Diseases, Hyperlipidemia complicated by chronic disease; CT, conventional therapy; /, not applicable.

### 3.8 Risk of publication bias

**FIGURE 8:**
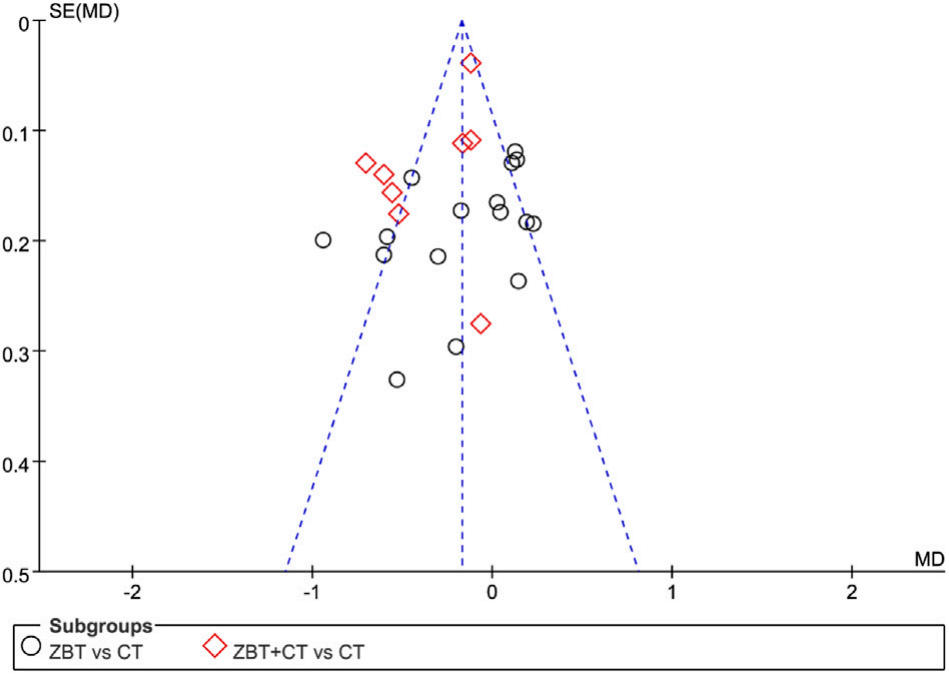
Funnel plot depicting the outcome of total cholesterol (TC). *MD*, mean difference; *se*, standard error; CT, conventional therapy.

By plotting funnel plots with the MD value for each publication, the horizontal coordinate of publication bias could be analyzed. The funnel plots revealed that the funnel was essentially inverted and asymmetric. The results implied the presence of publication bias. Figure 8 depicts a funnel plot of TC level.

### 3.9 Certainty of evidence

The certainty of the evidence was low (Table 6). The effective rate was low based on the results of TC, TG, LDL-C, and HDL-C. The primary grounds for downgrading were uncertainty regarding bias and inconsistency.

**TABLE 6:** Summary of the study findings.

Outcome	Certainty assessment						No of participants			Effect		Certainty	Importance
	No of studies	Study design	Risk of bias	Inconsistency	Indirectness	Imprecision	Other considerations	Continuous variables	Placebo	Relative (95% CI)	Absolute (95% CI)		
TG	25	RCT	Serious	Serious	Not serious	Not serious	None	1313	1313	—	MD 0.17 lower (0.31 lower to 0.04 lower)	⊕⊕○○	Important
												Low	
LDL-C	25	RCT	Serious	Serious	Not serious	Not serious	None	1313	1313	—	MD 0.16 lower (0.3 lower to 0.03 lower)	⊕⊕○○	Important
												Low	
HDL-C	22	RCT	Serious	Serious	Not serious	Not serious	None	1202	1203	—	MD 0.09 higher (0.02 higher to 0.15 higher)	⊕⊕○○	Important
												Low	
TC	25	RCT	Serious	Serious	Not serious	Not serious	None	1313	1313	—	MD 0.29 lower (0.45 lower to 0.12 lower)	⊕⊕○○	Important
												Low	
The effective rate	11	RCT	Serious	Serious	Not serious	Not serious	None	523/564 (92.7%)	481/563 (85.4%)	OR 2.32 (1.16–4.66)	77 more per 1,000 (from 18 more to 110 more)	⊕⊕○○	Important
												Low	

Abbreviations: CI, confidence interval; MD, mean difference.

## 4 Discussion

### 4.1 Summary of results

This systematic review included 2,952 participants from 28 RCTs and assessed the efficacy and safety of ZBT for the treatment of HLP. The efficacy and safety of ZBT or ZBT plus CT were evaluated in comparison to that of CT alone.

#### 4.1.1 Zhibitai compared with conventional therapy

Seventeen studies with a total of 1,753 participants reported outcomes with ZBT vs. CT treatment. A meta-analysis revealed that ZBT was as effective as CT for TG, TC, LDL-C, HDL-C, and total effective rate. To determine the impact of other factors on the efficacy of ZBT, a subgroup analysis was conducted using different treatment duration and different dosages of ZBT. The results showed that the ZBT group performed similarly to the control group, independent of the treatment duration (<8 weeks, ≥8 weeks), and the difference in interactive effect was significant in TC with different dosages of ZBT. In terms of safety, the incidence of advanced events in the ZBT group was lower than that in the control group, especially in liver functions, suggesting that ZBT may be safe. A clinical trial study confirmed this conclusion that the lipid lowing effect of intensive therapy with Chinese medicine ZBT in patients with moderate and high risk of atherosclerosis is the same as statins (Xu et al., 2010). Modern pharmacologic research found that the monacolin K recognized lipid-lowering qualities found in RYR had a similar structure to Statins (Endo, 1979) that primarily functions by inhibiting 3-hydroxy-3-methylglutaryl CoA (HMG CoA) reductase (Cicero et al., 2019). The extract of CPB can reduce blood lipid levels and protect the cardiovascular system (Wu et al., 2020; Zhang et al., 2020). AR and AMR passed the sirt1-lxr α- ABCA1 /sr-bⅠ pathway, which promotes reverse cholesterol transport, improves lipid metabolism, and prevents vascular thickening (Wei et al., 2021).

#### 4.1.2 Zhibitai plus conventional therapy compared with conventional therapy alone

Eleven studies, including 1199 participants, reported ZBT plus CT versus CT alone. The results of the meta-analysis showed that ZBT plus CT could significantly decrease the level of TC, TG, LDL-C and improve the level of HDL-C, than CT alone. The addition of ZBT therapy to CT resulted in higher total effective rate, notably decreasing complications. The results showed that ZBT has promoted the effect of CT for hyperlipidemia. ZBT might improve the efficacy of CT in reducing TC while increasing HDL. It could be attributable to an effect on very low-density lipoprotein (VLDL) (Nakajima et al., 2019). The results of subgroup analysis showed the combination of ZBT and CT performed better than CT alone in a subgroup of treatment duration. A limited sample size might be responsible for this deviation, a more detailed understanding of the specific mechanism is still needed. As for the safety profile, the results showed that the incidence of ZBT plus CT was lower than that of CT alone, especially in gastrointestinal reactions. It might be because the constituents of ZBT included AMR, CPB, and RYR, which can protect the gastrointestinal tract, and prevent gastrointestinal reactions (Sun et al., 2019; Wan et al., 2019; Li et al., 2020). Research also finds that the antioxidant potential of RYR preserved normal levels of ubiquinones in the heart, reduced the occurrence of atorvastatin related myopathy (Abdelbaset et al., 2014).

### 4.2 Risk of bias

Even though we try to avoid bias during the study, some limitations are inevitable. Randomization and blinding were not reported in the majority of the studies. The risk of selection and performance bias was difficult to assess. Since none of the included studies reported trial registration, the study process was not transparent. The overall bias of 85.71% of studies was classified as some concerns, with a high risk of 14.29%, which reduces reducing the credibility of the results. In addition, there were no studies that performed intention-to-treat analyses, which exaggerated the efficacy of ZBT. Therefore, the results of this review should be considered with caution.

### 4.3 Certainty of evidence

The GRADE approach assessed the certainty of the evidence in this review. The certainty of each outcome was low, which led to our cautious attitude of the results. The primary reasons for downgrading were the risk of bias and inconsistency. High-quality RCTs should be undertaken to improve the certainty of the evidence concerning ZBT in HLP.

### 4.4 Heterogeneity between the included studies

The cause for statistical heterogeneity among studies was clinical or methodological (Melsen et al., 2014). This review addressed clinical heterogeneity by designing rigorous eligibility criteria, including specific participant, intervention, comparison, outcomes, and study designs. In addition, this review conducted a subgroup analysis, based on ZBT plus CT or not, the duration of intervention, and the dosage of ZBT. However, there may be some inevitable clinical heterogeneity. First, there was a difference in the demographic characteristics of participants across the studies; however, it was difficult to distinguish across studies in terms of age, gender, and comorbidity information. Second, the intervention of the control group in this review was CT, although the details of CT were not described in each original study. Different studies might have different CTs, which may be a source of heterogeneity. This review conducted a sensitivity analysis in addition to subgroup analysis, albeit the source of heterogeneity could not be identified.

### 4.5 Clinical implications

The safety and efficacy of ZBT in the treatment of HLP, whether alone or in combination with CT, were investigated in this review. The outcomes were TG, TC, LDL-C, HDL-C, and total effective rate. The results showed that ZBT is similar to CT and that ZBT and CT combination performed better than CT alone in improving the levels of TG, TC, LDL-C, and HDL-C. The incidence of AEs was comparable between the two groups. As a result, ZBT may be a complementary and alternative medicine for HLP. When compared with people with normal TC levels, people with HLP showed about twice the risk of CVD (Writing Group et al., 2016). Therefore, ZBT can be potentially used as a complementary treatment to reduce CVD risk in primary care.

### 4.6 Strengths and limitations

This article is the first systematic review and meta-analysis of ZBT in the treatment of HPL. It includes a large sample size of 2,952 participants from 28 RCTs. A consistent trend emerged from both subgroup analysis and sensitivity analysis, which indicated that the results were stable. However, there are several limitations to this review. First, even after accounting for subgroups and sensitivity analyses, the meta-analysis of primary outcomes revealed significant heterogeneity. Second, the included studies were of low quality, citing “some concerns.” about bias in the majority of them, which reduced the credibility of the results. Third, no comparison of ZBT with other Chinese medicines was performed, and the specific efficacy of ZBT could not be evaluated comprehensively. Finally, there was a lack of information regarding the dosage of CT. As a result, it was difficult to establish the net benefit of ZBT when combined with different dosages of CT. These points should be explored in future research.

## 5 Conclusion

Based on current evidence, the efficacy of ZBT alone is equal to CT in terms of the treatment outcomes of HLP. Moreover, ZBT plus CT performed better than CT alone, indicating that ZBT may provide benefits to patients with HLP in a safe manner. In this study, the included studies showed a low level of quality, which reduced the credibility of the assessment. Thus, RCTs with high quality are urgently warranted.

## Data Availability

The original contributions presented in the study are included in the article/Supplementary Material, further inquiries can be directed to the corresponding authors.
