# Supplementary material for: Efficacy and safety of zhibitai in the treatment of hyperlipidemia: A systematic review and meta-analysis
Source: Front Pharmacol. 2022 Sep 2;13:974995. doi: 10.3389/fphar.2022.974995 (PMC9479062; doi:10.3389/fphar.2022.974995)
Supplement: Supplementary file 1 [file Table1.DOCX]

| Appendix I | | | | |
| --- | --- | --- | --- | --- |
| The results of the sensitivity analysis of the effect of Zhibitai (ZBT) vs conventional therapy in TG | | | | |
| **Study** | ***P*** | ***I^2^*** | **MD** | **95%CI** |
| Dan-yan Xu-2010 | 0.69267 | 90.42% | -0.05 | [-0.29;0.19] |
| Hu-2012 | 0.66915 | 90.53% | -0.05 | [-0.29;0.19] |
| Huang-2013 | 0.21906 | 85.52% | -0.11 | [-0.3;0.07] |
| He-2013 | 0.53999 | 89.90% | -0.07 | [-0.3;0.16] |
| Bai-2019 | 0.91558 | 90.30% | -0.01 | [-0.24;0.22] |
| Xiong-2019 | 0.67347 | 90.55% | -0.05 | [-0.29;0.19] |
| Zhou-2014 | 0.68649 | 90.60% | -0.05 | [-0.29;0.19] |
| Sun-2014 | 0.95946 | 85.60% | -0.01 | [-0.23;0.22] |
| Wang-2015 | 0.84918 | 90.63% | -0.02 | [-0.26;0.21] |
| Hua-2020 | 0.69795 | 90.58% | -0.05 | [-0.29;0.19] |
| Pang-2018 | 0.80961 | 90.70% | -0.03 | [-0.27;0.21] |
| Pang-2018 | 0.59165 | 89.58% | -0.06 | [-0.3;0.17] |
| Li-2007 | 0.62789 | 90.51% | -0.06 | [-0.29;0.18] |
| Bai-2010 | 0.83498 | 90.67% | -0.02 | [-0.26;0.21] |
| Zhu-2010 | 0.74059 | 90.70% | -0.04 | [-0.27;0.19] |
| Hao-2010 | 0.87569 | 89.74% | 0.02 | [-0.19;0.22] |

Supplementary Material

# Supplementary Tables

| The results of the sensitivity analysis of the effect of Zhibitai (ZBT) vs conventional therapy in TC | | | | |
| --- | --- | --- | --- | --- |
| **Study** | ***P*** | ***I^2^*** | **MD** | **95%CI** |
| Dan-yan Xu-2010 | 0.05436 | 73.57% | -0.18 | [-0.36;0] |
| Hu-2012 | 0.07971 | 74.77% | -0.17 | [-0.35;0.02] |
| Huang-2013 | 0.05969 | 73.92% | -0.17 | [-0.36;0.01] |
| He-2013 | 0.06746 | 73.79% | -0.17 | [-0.36;0.01] |
| Bai-2019 | 0.22852 | 62.51% | -0.09 | [-0.24;0.06] |
| Zhou-2014 | 0.0621 | 73.25% | -0.17 | [-0.36;0.01] |
| Zhou-2012 | 0.06325 | 73.14% | -0.17 | [-0.36;0.01] |
| Bai-2018 | 0.11489 | 75.02% | -0.15 | [-0.34;0.04] |
| Wang-2015 | 0.17545 | 71.92% | -0.12 | [-0.3;0.05] |
| Hua-2020 | 0.08251 | 74.83% | -0.16 | [-0.35;0.02] |
| Pang-2018 | 0.11019 | 75.05% | -0.15 | [-0.33;0.03] |
| Li-2020 | 0.16771 | 71.73% | -0.13 | [-0.31;0.05] |
| Li-2007 | 0.06967 | 74.60% | -0.17 | [-0.35;0.01] |
| Liu-2011 | 0.13073 | 74.68% | -0.14 | [-0.33;0.04] |
| Zhu-2010 | 0.17352 | 72.21% | -0.12 | [-0.3;0.05] |
| Hao-2013 | 0.13963 | 74.28% | -0.13 | [-0.31;0.04] |

| The results of the sensitivity analysis of the effect of Zhibitai (ZBT) vs conventional therapy in LDL-C | | | | |
| --- | --- | --- | --- | --- |
| **Study** | ***P*** | ***I^2^*** | **MD** | **95%CI** |
| Dan-yan Xu-2010 | 0.65249 | 89.78% | 0.05 | [-0.17;0.27] |
| Hu-2012 | 0.78307 | 89.64% | 0.03 | [-0.19;0.25] |
| Huang-2013 | 0.66634 | 89.81% | 0.05 | [-0.17;0.26] |
| He-2013 | 0.89521 | 88.71% | 0.01 | [-0.2;0.23] |
| Bai-2019 | 0.31232 | 83.17% | 0.09 | [-0.09;0.27] |
| Xiong-2019 | 0.86603 | 89.31% | 0.02 | [-0.2;0.23] |
| Zhou-2014 | 0.87223 | 89.34% | 0.02 | [-0.2;0.23] |
| Bai-2018 | 0.64931 | 89.79% | 0.05 | [-0.17;0.27] |
| Wang-2015 | 0.50763 | 89.16% | 0.07 | [-0.14;0.28] |
| Hua-2020 | 0.74139 | 89.76% | 0.04 | [-0.18;0.25] |
| Pang-2018 | 0.84217 | 89.55% | 0.02 | [-0.19;0.24] |
| Li-2020 | 0.68006 | 89.81% | 0.05 | [-0.17;0.26] |
| Li-2007 | 0.78701 | 89.64% | 0.03 | [-0.19;0.25] |
| Liu-2011 | 0.78050 | 86.78% | -0.03 | [-0.2;0.15] |
| Zhu-2010 | 0.85329 | 89.43% | 0.02 | [-0.19;0.23] |
| Hao-2010 | 0.52173 | 88.60% | 0.07 | [-0.14;0.28] |

| The results of the sensitivity analysis of the effect of Zhibitai (ZBT) vs conventional therapy in HDL-C | | | | |
| --- | --- | --- | --- | --- |
| **Study** | ***P*** | ***I^2^*** | **MD** | **95%CI** |
| Dan-yan Xu-2010 | 0.80304 | 93.37% | 0.01 | [-0.09;0.11] |
| Hu-2012 | 0.70451 | 93.31% | 0.02 | [-0.08;0.12] |
| Huang-2013 | 0.73589 | 93.36% | 0.02 | [-0.08;0.11] |
| He-2013 | 0.41565 | 87.07% | 0.04 | [-0.05;0.12] |
| Xiong-2019 | 0.74545 | 93.34% | 0.02 | [-0.08;0.11] |
| Zhou-2014 | 0.64145 | 93.19% | 0.02 | [-0.07;0.12] |
| Bai-2018 | 0.64935 | 85.57% | -0.02 | [-0.09;0.06] |
| Wang-2015 | 0.99321 | 93.15% | 0 | [-0.1;0.1] |
| Hua-2020 | 0.92989 | 93.31% | 0 | [-0.09;0.1] |
| Li-2020 | 0.97875 | 92.78% | 0 | [-0.1;0.09] |
| Li-2007 | 0.76077 | 93.36% | 0.02 | [-0.08;0.11] |
| Liu-2011 | 0.6903 | 93.34% | 0.02 | [-0.08;0.11] |
| Zhu-2010 | 0.58904 | 93.25% | 0.03 | [-0.07;0.12] |

| The results of the sensitivity analysis of the effect of Zhibitai (ZBT) vs conventional therapy in total effective rate | | | | |
| --- | --- | --- | --- | --- |
| **Study** | ***P*** | ***I^2^*** | **OR** | **95%CI** |
| Hu-2012 | 0.913 | 71.80% | 1.08 | [0.27;4.4] |
| He -2013 | 0.163 | 0% | 1.66 | [0.81;3.39] |
| Bai -2019 | 0.609 | 52.40% | 0.77 | [0.28;2.1] |
| Xiang-2013 | 0.948 | 71.60% | 1.05 | [0.27;4] |
| Li-2020 | 0.948 | 71.60% | 1.05 | [0.27;4] |

| The results of the sensitivity analysis of the effect of ZBT plus CT vs CT in TG | | | | |
| --- | --- | --- | --- | --- |
| **Study** | ***P*** | ***I^2^*** | **MD** | **95%CI** |
| Tan-2021 | ＜0.0001 | 46.12% | -0.34 | [-0.41;-0.27] |
| Shi-2019 | ＜0.0001 | 56.91% | -0.38 | [-0.51;-0.25] |
| Chen-2016 | ＜0.0001 | 52.05% | -0.4 | [-0.52;-0.28] |
| Xiong-2019 | ＜0.0001 | 55.03% | -0.4 | [-0.52;-0.27] |
| Sun-2014 | ＜0.0001 | 5.03% | -0.34 | [-0.41;-0.27] |
| Xiao-2020 | ＜0.0001 | 56.83% | -0.38 | [-0.51;-0.24] |
| Chen-2020 | ＜0.0001 | 57.11% | -0.39 | [-0.53;-0.24] |
| He-2020 | ＜0.0001 | 55.00% | -0.39 | [-0.52;-0.27] |
| Hao-2013 | ＜0.0001 | 54.20% | -0.39 | [-0.5;-0.27] |
| Tan-2021 | ＜0.0001 | 46.12% | -0.34 | [-0.41;-0.27] |
| Shi-2019 | ＜0.0001 | 56.91% | -0.38 | [-0.51;-0.25] |
| Chen-2016 | ＜0.0001 | 52.05% | -0.4 | [-0.52;-0.28] |
| Chen-2016 | ＜0.0001 | 55.03% | -0.4 | [-0.52;-0.27] |
| Sun-2014 | ＜0.0001 | 5.03% | -0.34 | [-0.41;-0.27] |
| Xiao-2020 | ＜0.0001 | 56.83% | -0.38 | [-0.51;-0.24] |
| Chen-2020 | ＜0.0001 | 57.11% | -0.39 | [-0.53;-0.24] |
| He-2020 | ＜0.0001 | 55.00% | -0.39 | [-0.52;-0.27] |
| Hao-2013 | ＜0.0001 | 54.20% | -0.39 | [-0.5;-0.27] |

| The results of the sensitivity analysis of the effect of ZBT plus CT vs CT in TC | | | | |
| --- | --- | --- | --- | --- |
| **Study** | ***P*** | ***I^2^*** | **MD** | **95%CI** |
| Tan-2021 | 0.00216 | 94.86% | -0.54 | [-0.88;-0.19] |
| Shi-2019 | 0.00175 | 94.81% | -0.54 | [-0.89;-0.2] |
| Chen-2016 | 0.00654 | 94.84% | -0.49 | [-0.85;-0.14] |
| Xiong-2019 | 0.00723 | 94.79% | -0.49 | [-0.84;-0.13] |
| Sun-2014 | 0.00018 | 80.65% | -0.35 | [-0.54;-0.17] |
| Xiao-2020 | 0.00788 | 94.71% | -0.48 | [-0.84;-0.13] |
| Chen-2020 | 0.00167 | 92.49% | -0.55 | [-0.89;-0.21] |
| He-2020 | 0.00912 | 94.48% | -0.47 | [-0.82;-0.12] |
| Ma-2018 | 0.00164 | 94.88% | -0.54 | [-0.87;-0.2] |

| The results of the sensitivity analysis of the effect of ZBT plus CT vs CT in LDL-C | | | | |
| --- | --- | --- | --- | --- |
| **Study** | ***P*** | ***I^2^*** | **MD** | **95%CI** |
| Tan-2021 | ＜0.0001 | 87.86% | -0.49 | [-0.65;-0.32] |
| Shi-2019 | ＜0.0001 | 88.22% | -0.51 | [-0.67;-0.34] |
| Chen-2016 | ＜0.0001 | 85.96% | -0.46 | [-0.61;-0.31] |
| Xiong-2019 | ＜0.0001 | 85.76% | -0.46 | [-0.62;-0.3] |
| Sun-2014 | ＜0.0001 | 85.53% | -0.46 | [-0.61;-0.3] |
| Wang-2015 | ＜0.0001 | 87.35% | -0.49 | [-0.66;-0.32] |
| Chen-2020 | ＜0.0001 | 21.50% | -0.55 | [-0.65;-0.44] |
| He-2020 | ＜0.0001 | 87.74% | -0.49 | [-0.66;-0.33] |
| Ma-2018 | ＜0.0001 | 88.31% | -0.49 | [-0.65;-0.33] |

| The results of the sensitivity analysis of the effect of ZBT plus CT vs CT in HDL-C | | | | |
| --- | --- | --- | --- | --- |
| **Study** | ***P*** | ***I^2^*** | **MD** | **95%CI** |
| Tan-2021 | ＜0.0001 | 77.78% | 0.14 | [0.08;0.2] |
| Shi-2019 | 0.00177 | 85.35% | 0.18 | [0.07;0.3] |
| Chen-2016 | 0.00018 | 86.06% | 0.2 | [0.1;0.31] |
| Xiong-2019 | 0.00037 | 86.76% | 0.2 | [0.09;0.31] |
| Sun-2014 | 0.00132 | 85.25% | 0.17 | [0.06;0.27] |
| Wang-2015 | 0.00156 | 87.08% | 0.18 | [0.07;0.3] |
| Chen-2020 | 0.00044 | 86.01% | 0.2 | [0.09;0.31] |
| He-2020 | 0.00086 | 87.43% | 0.19 | [0.08;0.31] |
| Ma-2018 | ＜0.0001 | 87.38% | 0.2 | [0.09;0.3] |

| The results of the sensitivity analysis of the effect of ZBT plus CT vs CT in total effective rate | | | | |
| --- | --- | --- | --- | --- |
| **Study** | ***P*** | ***I^2^*** | **OR** | **95%CI** |
| Pan -2021 | ＜0.0001 | 0% | 4.98 | [2.51;9.85] |
| Shi-2019 | ＜0.0001 | 0% | 3.9 | [2.04;7.45] |
| Chen -2016 | ＜0.0001 | 0% | 4.57 | [2.42;8.64] |
| Sun-2014 | ＜0.0001 | 0% | 4.85 | [2.52;9.34] |
| Chen-2020 | ＜0.0001 | 0% | 5.69 | [2.71;11.94] |
| Zhao -2013 | ＜0.0001 | 0% | 4.25 | [2.18;8.29] |
