# Supplementary material for: Efficacy and safety of zhibitai in the treatment of hyperlipidemia: A systematic review and meta-analysis
Source: Front Pharmacol. 2022 Sep 2;13:974995. doi: 10.3389/fphar.2022.974995 (PMC9479062; doi:10.3389/fphar.2022.974995)
Supplement: Supplementary file 2 [file Table2.DOCX]

Annex: Literature retrieval strategy

CNKI: SU %= 'zhibitai' AND (SU %= 'dyslipidemia' OR SU %= 'lipids' OR SU %='hyperlipidemia' OR SU %='triglycerides' OR SU %= 'total cholesterol' OR SU %= 'high-density lipoprotein' OR SU %= 'low density lipoprotein' OR SU %= 'safety' OR SU %= 'efficient')

Wanfang: ((topic: zhibitai) and ((topic: hyperlipidemia) or (topic: dyslipidemia) or (topic: lipids) or (topic: triglycerides) or (topic: total cholesterol) or (topic: high-density lipoprotein) or (topic: low density lipoprotein) or (topic: effective rate) or (topic: security))) and date: * - 2022

VIP: M = (hyperlipidemia OR dyslipidemia OR blood lipid OR triglyceride OR total cholesterol OR high-density lipoprotein OR low-density lipoprotein OR safety OR response rate) AND M= zhibitai

SinoMed: "zhibitai" AND "hyperlipidemia" OR "dyslipidemia" OR "total cholesterol" OR "triglyceride" OR "high density lipoprotein" OR "high density lipoprotein" "Low density lipoprotein" OR "Safety"

PubMed:

#1 Hyperlipidemias [MeSH Terms]

#2 Hyperlipemi [Title/Abstract]

#3 Hyperlipemias [Title/Abstract]

#4 hyperlipidemia [Title/Abstract]

#5 Lipidemia [Title/Abstract]

#6 Lipidemias [Title/Abstract]

#7 Lipemia [Title/Abstract]

#8 Lipemias [Title/Abstract]

#9 #1 OR #2 OR #3 OR #4 OR #5 OR #6 OR #7 OR #8

#10 Zhibitai [Title/Abstract]

#11 #9 AND #10

Embase:

#1. zhibitai: ti, ab, kw

#2. 'zhibitai'/exp

#3. #1 OR #2

#4. dyslipidemia: ti, ab, kw

#5 hyperlipemi: ti, ab, kw

#6 hyperlipemias: ti, ab, kw

#7 hyperlipidemia: ti, ab, kw

#8 lipidemia: ti, ab, kw

#9 lipidemias: ti, ab, kw

#10 lipemia: ti, ab, kw

#11 lipemias: ti, ab, kw

#12. 'hyperlipidemias'/exp

#13. #4 OR #5 OR #6 OR #7 OR #8 OR #9 OR #10 OR #11 OR #12

#14. #3 AND #13

Cochrane Library

#1. zhibitai: ti, ab, kw

#2. dyslipidemia: ti, ab, kw

#3 hyperlipemi: ti, ab, kw

#4 hyperlipemias: ti, ab, kw

#5 hyperlipidemia: ti, ab, kw

#6 lipidemia: ti, ab, kw

#7 lipidemias: ti, ab, kw

#8 lipemia: ti, ab, kw

#9 lipemias: ti, ab, kw

#10. MeSH descriptor: [hyperlipidemias] explode all trees

#11. #2 OR #3 OR #4 OR #5 OR #6 OR #7 OR #8 OR #9 OR #10

#12. #1 AND #11

Web of science

((TS= (zhibitai)) AND (((((((TS= (Hyperlipemi)) OR TS= (Hyperlipemias)) OR TS= (hyperlipidemia)) OR TS= (Lipidemia)) OR TS= (Lipidemias)) OR TS= (Lipemia)) OR TS= (Lipemias)))
