# Supplementary material for: Efficacy and safety of zhibitai in the treatment of hyperlipidemia: A systematic review and meta-analysis
Source: Front Pharmacol. 2022 Sep 2;13:974995. doi: 10.3389/fphar.2022.974995 (PMC9479062; doi:10.3389/fphar.2022.974995)
Supplement: Supplementary file 3 [file Table5.DOCX]

Zhibitai (ZBT) capsule (Chengdu Diao Jiuhong Pharmaceutical Factory, Chengdu, China), an oral Chinese patent medicine, was licensed as a drug used to treat hyperlipidemia by the National Medical Products Administration (NMPA) of China in May 2019 ([NMPA, 2019](#OLE_LINK80)). ZBT contains four medicinal herbs: Crataegus pinnatifida Bunge (CPB) [Rosaceae; [crataegi fructus](https://mpns.science.kew.org/mpns-portal/drugDetail?drugName=crataegi+fructus&query=Shanzha&filter=&fuzzy=false&nameType=all)], Alisma plantago-aquatica subsp. Orientale (Sam.) Sam. (AR) [Alismataceae; [alismatis rhizoma](https://mpns.science.kew.org/mpns-portal/drugDetail?drugName=alismatis+rhizoma&query=Zexie&filter=&fuzzy=false&nameType=all)], Atractylodes macrocephala Koidz (AMR) [Asteraceae; [atractylodis macrocephalae rhizoma](https://mpns.science.kew.org/mpns-portal/drugDetail?drugName=atractylodis+macrocephalae+rhizoma&query=Baizhu&filter=&fuzzy=false&nameType=all)], and red yeast rice (RYR) [Rice fermented by monascus]. According to the national drug standard of China, the content determination index of ZBT is ursolic acid and oleanolic acid (Ursolic acid ≥2.2mg/capsule) ([CMIP, 2022](#OLE_LINK2)). The other major component of ZBT is lovastatin, the content of it was ≥8.3mg/capsule ([Shi et al., 2019](#OLE_LINK3); [Fu et al., 1999](#OLE_LINK4); [Wen et al., 2011](#OLE_LINK5)).

Reference

China Medical Information Platform (CMIP) (2022). Content determination of zhibitai capsules. Available from <https://www.dayi.org.cn/drug/1146003> (Accessed August 9, 2022)

Fu, H. M., Song, H. T., Chen, L., Yang, G. J., and Guo, T. (1999). Study on the active components of reducing blood lipid in red yeast. *Chinese Traditional and Herbal Drugs.* 03.

National Medical Products Administration (NMPA) (2019). Domestic drug basic information query. Available from https://www.nmpa.gov.cn/datasearch/searchinfo.html?nmpa=aWQ9MTAyMDM4Jml0ZW1JZD1mZjgwODA4MTdjODMxMmM0MDE3YzliYmZjOGRlMDM2MA== (Accessed August 4, 2022).

Shi, L., Ye, J., and Song, L. (2019). Study on dissolution determination of zhibitai capsules. *Journal of Pharmaceutical Practice.* 37, 02.

Wen, Z. M., Zhang, F., Huang, Y., Xie, J., Luo, J. X., Wei, Z. X., et al. (2011). Determination of monascus in zhibitai capsules. *China Pharmaceuticals.* 20, 13.
