# Supplementary material for: Efficacy and safety of zhibitai in the treatment of hyperlipidemia: A systematic review and meta-analysis
Source: Front Pharmacol. 2022 Sep 2;13:974995. doi: 10.3389/fphar.2022.974995 (PMC9479062; doi:10.3389/fphar.2022.974995)
Supplement: Supplementary file 4 [file Table3.DOCX]

**Abbreviations**

| AEs | adverse events |
| --- | --- |
| ASCVD | arteriosclerotic cardiovascular disease |
| AR | Alisma plantago-aquatica subsp. orientale (Sam.) sam |
| AMR | Atractylodes macrocephala Koidz |
| C | control group |
| CPB | Crataegus pinnatifida Bunge |
| 95%CI | 95% confidence interval |
| CT | conventional therapy |
| CVD | cardiovascular disease |
| HDL-C | high-density lipoprotein cholesterol |
| HLP | hyperlipidemia |
| LDL-C | low-density lipoprotein cholesterol |
| MD | mean difference |
| OR | odds ratio |
| PRISMA | Preferred Reporting Items for Systematic Reviews and Meta-Analyses |
| RCTs | randomized controlled trials |
| RoB 2.0 | Cochrane risk-of-bias tool for randomized trials 2.0 |
| RR | risk ratio |
| RYR | red yeast rice |
| T | [treatment group](javascript:;) |
| TC | total cholesterol |
| TCM | traditional Chinese medicine |
| TG | triglyceride |
| VLDL | very low-density lipoprotein |
| ZBT | Zhibitai capsules |
