# Supplementary material for: Efficacy and safety of zhibitai in the treatment of hyperlipidemia: A systematic review and meta-analysis
Source: Front Pharmacol. 2022 Sep 2;13:974995. doi: 10.3389/fphar.2022.974995 (PMC9479062; doi:10.3389/fphar.2022.974995)
Supplement: Supplementary file 5 [file Table4.DOCX]

| **Patented formulations (Zhibitai** **capsule)** | | | | | |
| --- | --- | --- | --- | --- | --- |
| **Study** | **Formulation** | **Source** | **Species** | **Quality**  **control**  **reported?**  **(Y/N)** | **Chemical**  **analysis**  **reported? (Y/N)** |
| Li (2007) | Zhibitai capsule | Chengdu Diao jiuhong pharmaceutical factory | - Crataegus pinnatifida Bunge [Rosaceae] - Alisma plantago-aquatica subsp. Orientale (Sam.) Sam. [Alismataceae] - Atractylodes macrocephala Koidz [Asteraceae] - Red yeast rice [Rice fermented by monascus] | N | N |
| Xu et al. (2010) | Zhibitai capsule | Chengdu Diao jiuhong pharmaceutical factory | - Crataegus pinnatifida Bunge [Rosaceae] - Alisma plantago-aquatica subsp. Orientale (Sam.) Sam. [Alismataceae] - Atractylodes macrocephala Koidz [Asteraceae] - Red yeast rice [Rice fermented by monascus] | N | N |
| Zhu et al. (2010) | Zhibitai capsule | Chengdu Diao jiuhong pharmaceutical factory | - Crataegus pinnatifida Bunge [Rosaceae] - Alisma plantago-aquatica subsp. Orientale (Sam.) Sam. [Alismataceae] - Atractylodes macrocephala Koidz [Asteraceae] - Red yeast rice [Rice fermented by monascus] | N | N |
| Liu et al. (2011) | Zhibitai capsule | Chengdu Diao system Pharmaceutical Group Co. LTD | - Crataegus pinnatifida Bunge [Rosaceae] - Alisma plantago-aquatica subsp. Orientale (Sam.) Sam. [Alismataceae] - Atractylodes macrocephala Koidz [Asteraceae] - Red yeast rice [Rice fermented by monascus] | N | N |
| Hu et al. (2012) | Zhibitai capsule | Chengdu Diao jiuhong pharmaceutical factory | - Crataegus pinnatifida Bunge [Rosaceae] - Alisma plantago-aquatica subsp. Orientale (Sam.) Sam. [Alismataceae] - Atractylodes macrocephala Koidz [Asteraceae] - Red yeast rice [Rice fermented by monascus] | N | N |
| Zhou et al. (2012) | Zhibitai capsule | / | - Crataegus pinnatifida Bunge [Rosaceae] - Alisma plantago-aquatica subsp. Orientale (Sam.) Sam. [Alismataceae] - Atractylodes macrocephala Koidz [Asteraceae] - Red yeast rice [Rice fermented by monascus] | N | N |
| Hao (2013) | Zhibitai capsule | / | - Crataegus pinnatifida Bunge [Rosaceae] - Alisma plantago-aquatica subsp. Orientale (Sam.) Sam. [Alismataceae] - Atractylodes macrocephala Koidz [Asteraceae] - Red yeast rice [Rice fermented by monascus] | N | N |
| He (2013) | Zhibitai capsule | Chengdu Diao jiuhong pharmaceutical factory | - Crataegus pinnatifida Bunge [Rosaceae] - Alisma plantago-aquatica subsp. Orientale (Sam.) Sam. [Alismataceae] - Atractylodes macrocephala Koidz [Asteraceae] - Red yeast rice [Rice fermented by monascus] | N | N |
| Huang et al. (2013) | Zhibitai capsule | / | - Crataegus pinnatifida Bunge [Rosaceae] - Alisma plantago-aquatica subsp. Orientale (Sam.) Sam. [Alismataceae] - Atractylodes macrocephala Koidz [Asteraceae] - Red yeast rice [Rice fermented by monascus] | N | N |
| Xiang (2013) | Zhibitai capsule | Chengdu Diao system Pharmaceutical Group Co. LTD | - Crataegus pinnatifida Bunge [Rosaceae] - Alisma plantago-aquatica subsp. Orientale (Sam.) Sam. [Alismataceae] - Atractylodes macrocephala Koidz [Asteraceae] - Red yeast rice [Rice fermented by monascus] | N | N |
| Zhao (2013) | Zhibitai capsule | Chengdu Diao jiuhong pharmaceutical factory | - Crataegus pinnatifida Bunge [Rosaceae] - Alisma plantago-aquatica subsp. Orientale (Sam.) Sam. [Alismataceae] - Atractylodes macrocephala Koidz [Asteraceae] - Red yeast rice [Rice fermented by monascus] | N | N |
| Sun et al. (2014) | Zhibitai capsule | Chengdu Diao jiuhong pharmaceutical factory | - Crataegus pinnatifida Bunge [Rosaceae] - Alisma plantago-aquatica subsp. Orientale (Sam.) Sam. [Alismataceae] - Atractylodes macrocephala Koidz [Asteraceae] - Red yeast rice [Rice fermented by monascus] | N | N |
| Zhou (2014) | Zhibitai capsule | / | - Crataegus pinnatifida Bunge [Rosaceae] - Alisma plantago-aquatica subsp. Orientale (Sam.) Sam. [Alismataceae] - Atractylodes macrocephala Koidz [Asteraceae] - Red yeast rice [Rice fermented by monascus] | N | N |
| Wang et al. (2015) | Zhibitai capsule | Chengdu Diao jiuhong pharmaceutical factory | - Crataegus pinnatifida Bunge [Rosaceae] - Alisma plantago-aquatica subsp. Orientale (Sam.) Sam. [Alismataceae] - Atractylodes macrocephala Koidz [Asteraceae] - Red yeast rice [Rice fermented by monascus] | N | N |
| Chen et al. (2016) | Zhibitai capsule | Chengdu Diao jiuhong pharmaceutical factory | - Crataegus pinnatifida Bunge [Rosaceae] - Alisma plantago-aquatica subsp. Orientale (Sam.) Sam. [Alismataceae] - Atractylodes macrocephala Koidz [Asteraceae] - Red yeast rice [Rice fermented by monascus] | N | N |
| Bai (2018) | Zhibitai capsule | Chengdu Diao jiuhong pharmaceutical factory | - Crataegus pinnatifida Bunge [Rosaceae] - Alisma plantago-aquatica subsp. Orientale (Sam.) Sam. [Alismataceae] - Atractylodes macrocephala Koidz [Asteraceae] - Red yeast rice [Rice fermented by monascus] | N | N |
| Ma et al. (2018) | Zhibitai capsule | Chengdu Diao jiuhong pharmaceutical factory | - Crataegus pinnatifida Bunge [Rosaceae] - Alisma plantago-aquatica subsp. Orientale (Sam.) Sam. [Alismataceae] - Atractylodes macrocephala Koidz [Asteraceae] - Red yeast rice [Rice fermented by monascus] | N | N |
| Pang. (2018) | Zhibitai capsule | Chengdu Diao jiuhong pharmaceutical factory | - Crataegus pinnatifida Bunge [Rosaceae] - Alisma plantago-aquatica subsp. Orientale (Sam.) Sam. [Alismataceae] - Atractylodes macrocephala Koidz [Asteraceae] - Red yeast rice [Rice fermented by monascus] | N | N |
| Bai (2019) | Zhibitai capsule | Chengdu Diao jiuhong pharmaceutical factory | - Crataegus pinnatifida Bunge [Rosaceae] - Alisma plantago-aquatica subsp. Orientale (Sam.) Sam. [Alismataceae] - Atractylodes macrocephala Koidz [Asteraceae] - Red yeast rice [Rice fermented by monascus] | N | N |
| Shi et al. (2019) | Zhibitai capsule | Chengdu Diao jiuhong pharmaceutical factory | - Crataegus pinnatifida Bunge [Rosaceae] - Alisma plantago-aquatica subsp. Orientale (Sam.) Sam. [Alismataceae] - Atractylodes macrocephala Koidz [Asteraceae] - Red yeast rice [Rice fermented by monascus] | N | N |
| Xiong (2019) | Zhibitai capsule | Chengdu Diao jiuhong pharmaceutical factory | - Crataegus pinnatifida Bunge [Rosaceae] - Alisma plantago-aquatica subsp. Orientale (Sam.) Sam. [Alismataceae] - Atractylodes macrocephala Koidz [Asteraceae] - Red yeast rice [Rice fermented by monascus] | N | N |
| Chen et al. (2020) | Zhibitai capsule | Chengdu Diao jiuhong pharmaceutical factory | - Crataegus pinnatifida Bunge [Rosaceae] - Alisma plantago-aquatica subsp. Orientale (Sam.) Sam. [Alismataceae] - Atractylodes macrocephala Koidz [Asteraceae] - Red yeast rice [Rice fermented by monascus] | N | N |
| He (2020) | Zhibitai capsule | Chengdu Diao jiuhong pharmaceutical factory | - Crataegus pinnatifida Bunge [Rosaceae] - Alisma plantago-aquatica subsp. Orientale (Sam.) Sam. [Alismataceae] - Atractylodes macrocephala Koidz [Asteraceae] - Red yeast rice [Rice fermented by monascus] | N | N |
| Hua (2020) | Zhibitai capsule | Chengdu Diao jiuhong pharmaceutical factory | - Crataegus pinnatifida Bunge [Rosaceae] - Alisma plantago-aquatica subsp. Orientale (Sam.) Sam. [Alismataceae] - Atractylodes macrocephala Koidz [Asteraceae] - Red yeast rice [Rice fermented by monascus] | N | N |
| Li et al. (2020) | Zhibitai capsule | Chengdu Diao jiuhong pharmaceutical factory | - Crataegus pinnatifida Bunge [Rosaceae] - Alisma plantago-aquatica subsp. Orientale (Sam.) Sam. [Alismataceae] - Atractylodes macrocephala Koidz [Asteraceae] - Red yeast rice [Rice fermented by monascus] | N | N |
| Xiao (2020) | Zhibitai capsule | Chengdu Diao jiuhong pharmaceutical factory | - Crataegus pinnatifida Bunge [Rosaceae] - Alisma plantago-aquatica subsp. Orientale (Sam.) Sam. [Alismataceae] - Atractylodes macrocephala Koidz [Asteraceae] - Red yeast rice [Rice fermented by monascus] | N | N |
| Pan (2021) | Zhibitai capsule | Chengdu Diao jiuhong pharmaceutical factory | - Crataegus pinnatifida Bunge [Rosaceae] - Alisma plantago-aquatica subsp. Orientale (Sam.) Sam. [Alismataceae] - Atractylodes macrocephala Koidz [Asteraceae] - Red yeast rice [Rice fermented by monascus] | N | N |
| Tan et al. (2021) | Zhibitai capsule | Chengdu Diao jiuhong pharmaceutical factory | - Crataegus pinnatifida Bunge [Rosaceae] - Alisma plantago-aquatica subsp. Orientale (Sam.) Sam. [Alismataceae] - Atractylodes macrocephala Koidz [Asteraceae] - Red yeast rice [Rice fermented by monascus] | N | N |
